# Pollen Tube and Plant Reproduction

**DOI:** 10.3390/ijms20030531

**Published:** 2019-01-27

**Authors:** Giampiero Cai, Stefano Del Duca

**Affiliations:** 1 Department of Life Sciences, University of Siena, 53100 Siena, Italy; 2 Department of Biological, Geological and Environmental Sciences, Alma Mater Studiorum University of Bologna, 40126 Bologna, Italy; stefano.delduca@unibo.it

The pollen tube was a fundamental step forward in the evolution of terrestrial plants; in fact, it allowed plants to liberate themselves from water demand during reproduction. The importance of this simple organism (the plant gametophyte) is also due to its peculiar cytological structure, characterized by tip growth and a cell wall that is not canonically structured but perfectly adapted to the growth mechanism. Being both a critical factor in sexual reproduction and an excellent cell model has made the pollen tube attractive to many researchers around the world. The growth of pollen tubes is a complex mechanism that summarizes the basic machineries of cellular expansion: polysaccharides and enzymes are transported by secretory vesicles to a specific location, a set of molecules and proteins translate external signals that influence the cytoskeleton of pollen tubes and thus its growth direction. This process is also sometimes subjected to regulatory mechanisms intended to allow the growth of “non-self” pollen tubes, and the removal of “self” pollen tubes. In this way, plants have been able to promote genetic variability, which is the essential basis of evolution. In this special issue, the process of pollen tube growth, including both the mechanisms that promote growth and those that regulate it, has been discussed through several contributions.

The ability of pollen to fertilize pistils is important for the reproduction of plants both of ornamental interest, such as Petunia, and of agronomic interest with a consequent impact also from the economic point of view. To understand the mechanisms of anther development, Yue et al. reconstructed the transcriptome and metabolome of petunia anthers at different developmental stages [1]. The authors found many differentially expressed genes involved in photosynthesis and metabolism of starch and sucrose, suggesting that the metabolic pathways of carbohydrates gradually decrease during anther development. The formation of pollen would be meaningless if not followed by the formation of the pollen tube, which is necessary for the transport of sperm. This tip-growth process requires a precise and synchronized organization of actin bundles. In their review, Zhang et al. summarized the distinct organization of actin in pollen tubes, with emphasis on the short but dynamic actin filaments in the apex, and on proteins that can regulate their dynamics [2]. Full knowledge of actin filaments is important for understanding a multitude of events that occur in the pollen tube, including the process of self-incompatibility. Once the pollen grain reaches a receptive stigma and the pollen tube begins to grow, it must endure a long journey with the aim of transporting the two sperm cells. The long journey of the pollen tube through the stigma, the style, the funiculus, and the micropyle is the subject of the review by Zheng et al., where the authors emphasized on accurate regulation and molecular dialogue necessary for fertilization [3]. Knowledge of these interactions is fundamental, in addition to basic research, for the control of the fertilization process. The natural control of fertilization is represented, among other things, by the mechanism of self-incompatibility. This is an efficient barrier to reproduction and a mechanism for promoting crossbreeding between genetically different plants, but it is a limiting factor when it comes to plants of agricultural interest. Many chrysanthemum cultivars are self-incompatible, and therefore it is difficult to generate pure lines that are important in breeding programs. Even in self-compatible lines, seed production is extremely variable, so much so that it is necessary to analyze the factors that cause differences in the seed set. Wang et al. analyzed the morphology of pollen, its germination rate, pistil receptivity, and embryo development, as well as transcriptomic profiles of mature stigmas and anthers [4]. The authors concluded that differences in fertility between progeny are due to differences in pollen germination rates and pistil receptivity. Fruit-bearing plants of agronomic interest such as apricots have a self-incompatibility mechanism based on S-RNases whose gene characterization is of undoubted importance. As described by Herrera et al., it is critical to find the various S-alleles of plants such as apricots, assigning the self-incompatible genotypes to self-incompatible groups, for the development of future breeding programs [5]. In other species, the contribution of the self-incompatibility process is even more difficult to understand. In the Orchidaceae genera, most Dendrobium species are self-incompatible but little is known of it, so much so that Niu et al. asked themselves how many phenotypes exist in Dendrobium and how they are distributed phylogenetically [6]. Their study showed a high number of phenotypes, suggesting the presence of many self-incompatibility determinants that have evolved recently. Information is also lacking on the mechanism of self-incompatibility in Primula. Lu et al. analyzed pollen germination and fructification in the case of self and non-self crosses, and evaluated the levels of transcriptomics [7]. In addition to differences in the growth ability of pollen tubes, transcriptomic analyses showed a high number of differentially expressed genes, including genes associated with carbohydrate metabolism and environmental adaptation. Self-incompatibility is a complex process that needs the co-participation of many molecules. In Malinae (a subtribe of Rosaceae), gametophytic self-incompatibility needs the production of female determinants (S-RNases) that must interact with male determinants. In addition to this basic mechanism, other proteins such as transglutaminase may be needed. Del Duca et al. hypothesize that transglutaminase can post-translationally modify several other proteins including actin and tubulin, thereby destabilizing the cytoskeleton, and inhibiting the growth of pollen tubes [8]. Finally, it is also necessary to reconsider the effects that environmental pollutants have on the reproductive process of plants by altering specific aspects. Ismael et al. analyzed cadmium because it is highly toxic to pollen and, more importantly, to understand the defense mechanisms [9]. The authors observed that selenium and molybdenum can counteract the absorption, translocation, and impact of cadmium on pollen grains of Brassica napus. This study can be a valuable support for improving plant fertility and thus increasing plant biomass under stressful conditions.
